# Pathophysiological Drivers of Mast Cell Activation Syndrome and Implications for Treatment

**DOI:** 10.26502/fjhs.427

**Published:** 2026-07-22

**Authors:** Spencer Collins, Tyler Williams, Edgar Sanchez, Devendra K. Agrawal

**Affiliations:** Department of Translational Research, College of Osteopathic Medicine of the Pacific, Western University of Health Sciences, Pomona, California 91766 USA

**Keywords:** Acalabrutinib, Bruton tyrosine kinase inhibitors, Cromolyn sodium, Corticosteroids, Cyclosporine, FcεRI, Histamine, Ibrutinib, IgE-Mediated Immune response, JAK/STAT, KIT(D816V), Ketotifen, Leukotrienes, Mast Cells, Mast Cell Activation Syndrome, Mastocytosis, Montelukast, Multisystemic, Non-IgE Activation, H1/H2 blockers, Heparin, Omalizumab, Prostaglandins, Tryptase, Zanubrutinib

## Abstract

Mast Cell Activation Syndrome (MCAS) is an underrecognized multisystem disorder causing nonspecific and diverse symptoms, making it difficult for clinicians to diagnose and treat. This comprehensive review provides an overview of the signs and symptoms of MCAS while investigating the known pathophysiological signaling pathways and mediators that contribute to mast cell (MC) dysregulation. Unraveling the mechanisms of MC activation is essential for elucidating the underlying disease and exploring techniques to improve quality of life. Immunoglobulin E (IgE)- and non-IgE-mediated pathways are emphasized in addition to the various intracellular signaling like PI3K/Akt/mTOR, RAS/MAPK, and JAK/STAT that play pivotal roles in the amplification of MC activation and dysregulation. After activation of intracellular pathways, MC degranulation releases mediators, notably histamine, tryptase, heparin, leukotrienes, prostaglandins, and cytokines, thus facilitating further aberrant regulation. Finally, with a thorough understanding of these advanced molecular processes, there are numerous opportunities to be able to apply specialized inhibitors and novel therapies for treating MCAS. With antihistamines remaining the most well-established first-line treatment, the goal is to continue strengthening research in the field through education on the immunology of MCAS and to encourage discoveries that improve diagnostic biomarkers and therapeutic strategies.

## 1. Introduction

Mast cells (MCs) were discovered by Paul Ehrlich in 1878 during a doctoral thesis, where he described them as having distinct purple intracellular granules when stained with aniline blue dye [1]. They have a highly complex and multifunctional relationship with the innate immune system, closely involved in activating the adaptive immune system. It has been largely accepted that MCs have an association with allergic and antiparasitic responses. Although this is a core idea, it is very oversimplified; these unique cells are integral to phagocytosis, cytokine release, and the release of vasoactive substances such as histamine, which promotes vasodilation and vascular permeability during inflammation [2]. Beyond these immunological functions, emerging evidence suggests the growing relevance of MCs in tissue homeostasis, fibrosis, and angiogenesis [3]. These extraordinary cells originate in bone marrow progenitors, relocate to the periphery, where they mature and differentiate, and are tightly regulated by their key surface receptors [4].

There are primarily two mechanisms of MC activation: IgE-dependent and IgE-independent pathways. IgE-dependent signaling occurs through engagement of the key surface receptor known as the fragment crystallizable epsilon receptor I (FCεRI) [5]. This receptor is fundamental to the T helper cell 2 (Th2) response and hypersensitivity responses [6–9]. In contrast, IgE-independent pathways occur independently of the classical Th2-IgE-FCεRI axis and involve numerous alternative receptors and signaling pathways.

Similarly to well-known professional antigen-presenting dendritic cells, MCs express pattern recognition receptors (PRRs), including Toll-like receptors (TLRs), that are specialized in detecting and binding pathogen-associated molecular patterns (PAMPs) as part of the innate immune system. Depending on the type of pathogen, whether a Gram-negative bacterium, Gram-positive bacterium, virus, fungus, or parasite, different PRRs recognize specific PAMPs, allowing MCs to coordinate with the complement cascade and other immune components, including basophils, eosinophils, T cells, and IgE, to generate an effective immune response [10–14]. With the widespread functions of MCs and the multitude of poorly understood aspects of their biology, the consequences of dysregulation and aberrant activation can lead to detrimental pathologies, including MCAS. This review explores the diverse mechanisms of MC activation and the complex signaling pathways and mediator effects that contribute to disease pathophysiology.

## 2. Mast Cell Activation Syndrome and Clinical Relevance

Mast cell activation syndrome (MCAS) is characterized by significant clinical and biological heterogeneity, reflecting the diverse mechanisms and mediators involved in MC activation. One of the defining characteristics of MCs is their ability to respond to an extensive range of environmental exposures, infections, medications, physical stimuli, hormonal changes, and dietary factors. Researchers define MCAS as a widespread reaction to a broad array of stimuli that trigger MCs [15]. These symptoms tend to be severe, recurrent, and commonly associated with comorbidities such as Ehlers-Danlos Syndrome (EDS) and Postural Orthostatic Tachycardia Syndrome (POTS). Although MCAS has historically been challenging to diagnose, there has been an increasing number of reported cases, raising the question of whether this reflects a true increase in disease prevalence, improved recognition of MCAS and its associated comorbidities, or advances in diagnostic methods.

Given its clinical and biological heterogeneity, MCAS is classified into three major subtypes. Primary MCAS is typically clonal and is mainly due to various types of KIT mutations, most commonly KIT (D816V). Such mutations have been associated with cutaneous mastocytosis, systemic mastocytosis (SM), and monoclonal MCAS [15]. Conversely, secondary MCAS is non-clonal and results from normal MCs reacting to specific triggers such as allergies, infections, chronic autoimmune diseases, and even neoplastic disorders [16–20]. Idiopathic MCAS is also non-clonal, with no known trigger, KIT mutation, or underlying condition. Although they present with similar clinical features, SM is distinguished from MCAS because it is always clonal, meaning that an abnormally increased number of MCs accumulate within tissues. In contrast, MCAS has a normal number of MCs, but they are hyperreactive or dysregulated.

Despite their differing etiologies, both MCAS and SM take a substantial toll on patients' quality of life. Studies have observed the negative implications of these conditions on health-related quality of life (HRQOL), along with the impact of health literacy on coping with the burden of disease [21]. Using European Organization for Research and Treatment of Cancer questionnaires together with ANOVA and linear regression analyses, the study found that both SM and MCAS were associated with significantly reduced HRQOL, whereas higher levels of disease-related knowledge were associated with modest improvements in HRQOL [21]. These findings emphasize the importance of expanding our understanding of the etiology and pathophysiology of MCAS to ultimately optimize patient outcomes. The burden on HRQOL can be attributed to the widespread involvement of multiple organ systems, including the dermatologic, cardiovascular, gastrointestinal, neurologic, and respiratory systems. The most common signs and symptoms that can be difficult to manage daily include abdominal pain, diarrhea, dermatographism, headache, flushing, impaired concentration and memory, and naso-ocular symptoms [22]. These symptoms may be constant or flare in response to triggers, depending on the type of MCAS.

## 3. Clinical Manifestations of MCAS and Disease Burden

**Figure 1:**
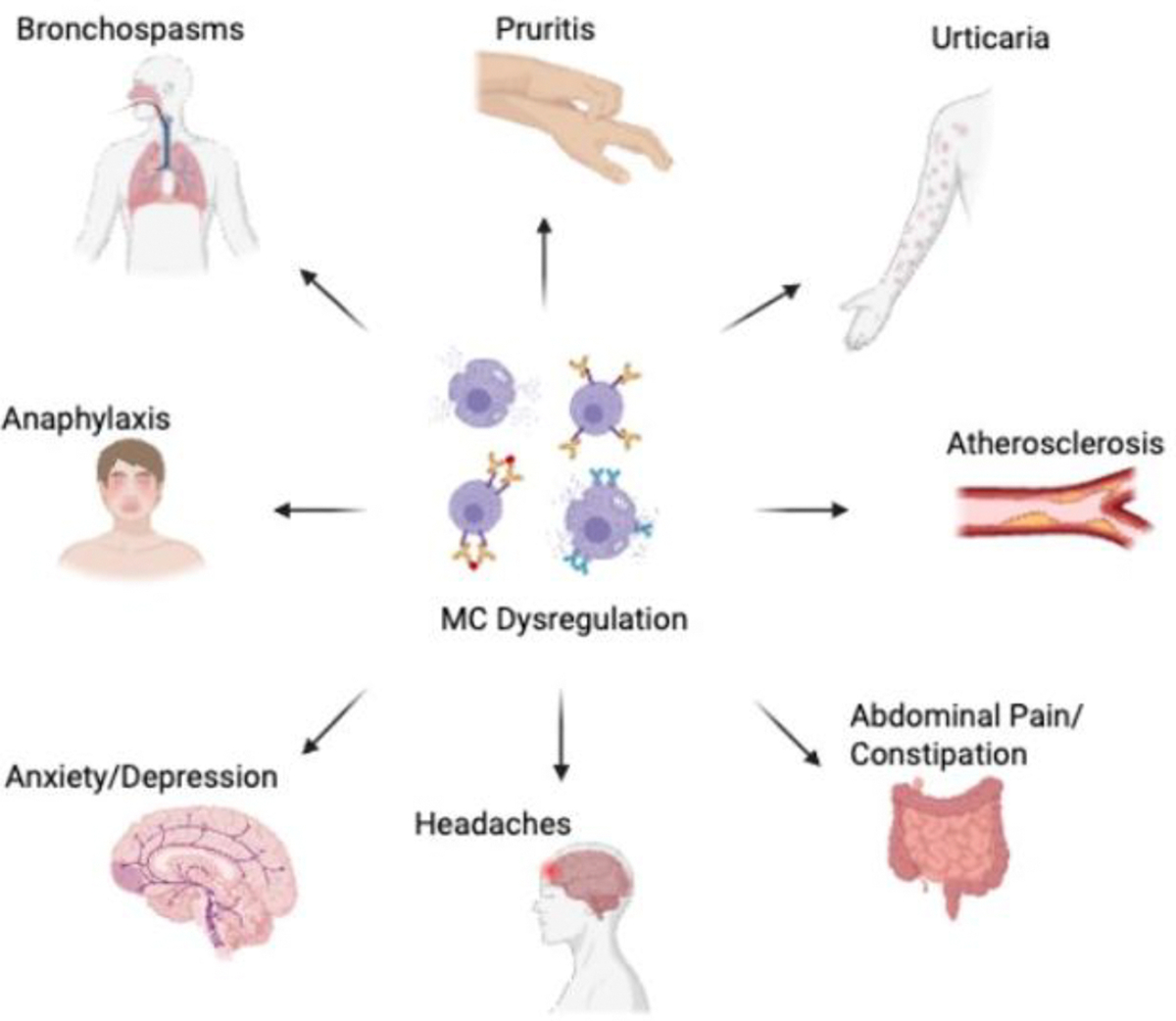
Representative systemic manifestations associated with MC dysregulation. MCAS is a multisystem disorder that can affect numerous organ systems, resulting in manifestations such as urticaria, pruritus, bronchospasm, abdominal pain, constipation, headaches, anxiety, depression, anaphylaxis, and cardiovascular disorders including atherosclerosis. MC, mast cells. Created with BioRender.com.

MCAS is a multisystem disorder that can affect numerous organ systems, including skin, cardiovascular, gastrointestinal, respiratory, neurologic, and others. Depending on its effect on an individual organ system, MCAS may present with a wide range of clinical symptoms, including skin reactions, severe allergic reactions, chronic fatigue, cognitive dysfunction, headaches, mood disturbance, gastrointestinal issues, and respiratory problems (Figure 1). Clinical manifestations due to MCAS in various organ systems is discussed in the following sections.

## 4. Dermatologic Manifestations due to MCAS

MCs are well recognized for their central role in allergic reactions, with dermatologic manifestations such as urticaria and flushing often among the first symptoms associated with MCAS and related disorders. This highlights the important role of dermatologists in distinguishing these multifaceted disorders to facilitate appropriate diagnosis and management. A review of the PubMed and Scopus databases investigated the dermatologic manifestations of idiopathic MCAS with the goal of strengthening the diagnostic acuity of dermatologists. Across 562 patients, dermatologic manifestations included bruising, dermatographism, unspecified rash, edema, sweating, and flushing [23]. These findings illustrate the wide range of cutaneous manifestations that MCAS can present with, emphasizing the importance of not overlooking these nonspecific signs and symptoms despite the relative rarity of the disorder. Beyond the role of MCs in allergic responses, clonal or non-clonal overactivation has been associated with chronic inflammation, excessive fibrosis, and keloid scarring, and in orthopedic dermatopathologies [24,25]. The wide variation in dermatologic symptoms contributes to the ongoing controversy surrounding the diagnostic criteria for MCAS and complicates both diagnosis and treatment. Consequently, individualized management plans are essential to address each patient's specific clinical presentation. As illustrated, dermatologic manifestations can be particularly burdensome and substantially impact quality of life, highlighting the importance of recognizing the broad spectrum of cutaneous presentations.

## 5. Cardiovascular manifestations due to MCAS

MC activation causes a release of numerous vasodilatory and pro-inflammatory mediators, including tryptase, cytokines, prostaglandins, and histamine, that underlie many of the cardiovascular manifestations of MCAS. The combined effects of these mediators promote hypotension, tachycardia, syncope, and vascular disease. Histamine plays a key role in the development of hypotension and tachycardia, as it increases vascular permeability, stimulates prostaglandin production, reduces myocardial contractility through H1 receptors, and induces positive chronotropy through H2 receptors [24]. Additional mediators, including tryptase, heparin, IL-8, IL-6, PGE2, and nitric oxide, contribute to vasodilation, leading to presyncope and syncope. In chronic cases, these mediators have also been associated with atherosclerosis through leukocyte recruitment and enhanced lipid uptake [26]. Preclinical mouse models have provided evidence that MCs contribute to the development of both atherosclerosis and postoperative restenosis following percutaneous coronary intervention or coronary artery bypass grafting. Researchers mimicked these procedures in the femoral arteries of mice using the murine arterial wire injury model to compare wild-type and MC-deficient mice. They observed pronounced neointimal hyperplasia, characterized by proliferation of the tunica intima resulting in luminal narrowing in wild-type mice, whereas this response was absent in MC-deficient counterparts [27]. Accumulation of evidence indicates that MCs play a crucial role in the process of attracting immune cells and proliferation of endothelial cells. These processes may further exacerbate the cardiovascular complications experienced by patients with excessive MC activation, and those with SM.

## 6. Gastrointestinal manifestations due to MCAS

Until recently, patients presenting with gastrointestinal (GI) symptoms associated with traditionally “allergic” disorders were overlooked and often dismissed. Gastroenterologists have increasingly recognized that the systemic effects of MC activation extend to the gut-brain axis, leading to a wide range of GI manifestations. Awareness of the commonly reported triad of MCAS, EDS, and POTS is warranted and may aid in the evaluation and management of GI symptoms. Patients frequently report GI symptoms including nausea, diarrhea, constipation, bloating, and abdominal pain. MC infiltration of the lamina propria, along with effects on nearby capillaries and submucosal neurons, has been implicated in the development of these symptoms [28]. Heightened histamine release has also been linked to idiopathic abdominal pain and visceral hypersensitivity, with elevated histamine and tryptase levels observed in affected individuals. Constipation and sensation of bloating may result from MC accumulation, which activates the immune system, alters the gut microbiome, and contributes to GI dysmotility [29]. Given the extensive role of the gut in systemic physiology, alterations in the diversity of the gut microbiome and its resident microorganisms may influence the gut-brain axis. These alterations may indirectly modulate vagal signaling, contributing to the neurologic manifestations of MCAS. Understanding these interconnected GI manifestations and their associated comorbidities may facilitate earlier diagnosis and more individualized management.

## 7. Neurological manifestations due to MCAS

The neurologic and psychiatric manifestations associated with MCAS have identifiable underlying mechanisms involving MC activation and neuroinflammation. MCAS may present with neurologic manifestations such as headache and dysautonomia, as well as psychiatric disorders including depression, generalized anxiety disorder, attention-deficit hyperactivity disorder, obsessive-compulsive disorder, phobias, and bipolar disorder [29]. Neuroinflammatory responses involving MCs occur within both the peripheral and central nervous systems. MC accumulation has been identified within the meninges, particularly the dura mater, as well as in blood vessels and around peripheral neurons. When activated, these MCs release inflammatory mediators that increase blood-brain barrier (BBB) permeability and compromise its integrity [30]. Disruption of the BBB heightens susceptibility to infection, inflammation, trauma, and psychological stress, which may lead to the development of neuropsychiatric manifestations. Mechanistically, headaches may result from the vasodilatory response induced by neuropeptides released within the meninges. These neuropeptides stimulate the autonomic nervous system, causing nociception by sensitizing the trigeminal nerve afferents [31]. Although neuropsychiatric manifestations are less common in MCAS, they can be debilitating and emphasize the need to identify the underlying cause.

## 8. Respiratory manifestations due to MCAS

Although MCAS has a classical association with anaphylaxis, activation of MCs induces bronchospasm, smooth muscle constriction, and other respiratory manifestations through the release of inflammatory mediators. Bioaerosols may also activate mast cells and the released mediators may activate other immune cells and receptors to trigger severe immune response [32–41]. During chronic respiratory disease, MCs enhance arachidonic acid metabolism through the cyclooxygenase pathway, generating prostaglandins and thromboxanes, whereas the lipoxygenase pathway produces leukotrienes, which are the more potent inflammatory mediators generated by these pathways [42]. Leukotrienes contribute strongly to bronchoconstriction and the development of obstructive respiratory diseases such as asthma. The specific leukotriene B4 is a strong chemoattractant thus recruiting local leukocytes to the site. Whereas sulfidopeptide leukotrienes C4, D4, and E4 are highly spasmogenic, resulting in constriction of smooth muscle and airway narrowing [43–45]. The combined effects of these mediators can produce wheezing, dyspnea, cough, and asthma exacerbations through airway inflammation and bronchoconstriction.

## 9. Mechanistic Drivers of Mast Cell Activation

**Figure 2:**
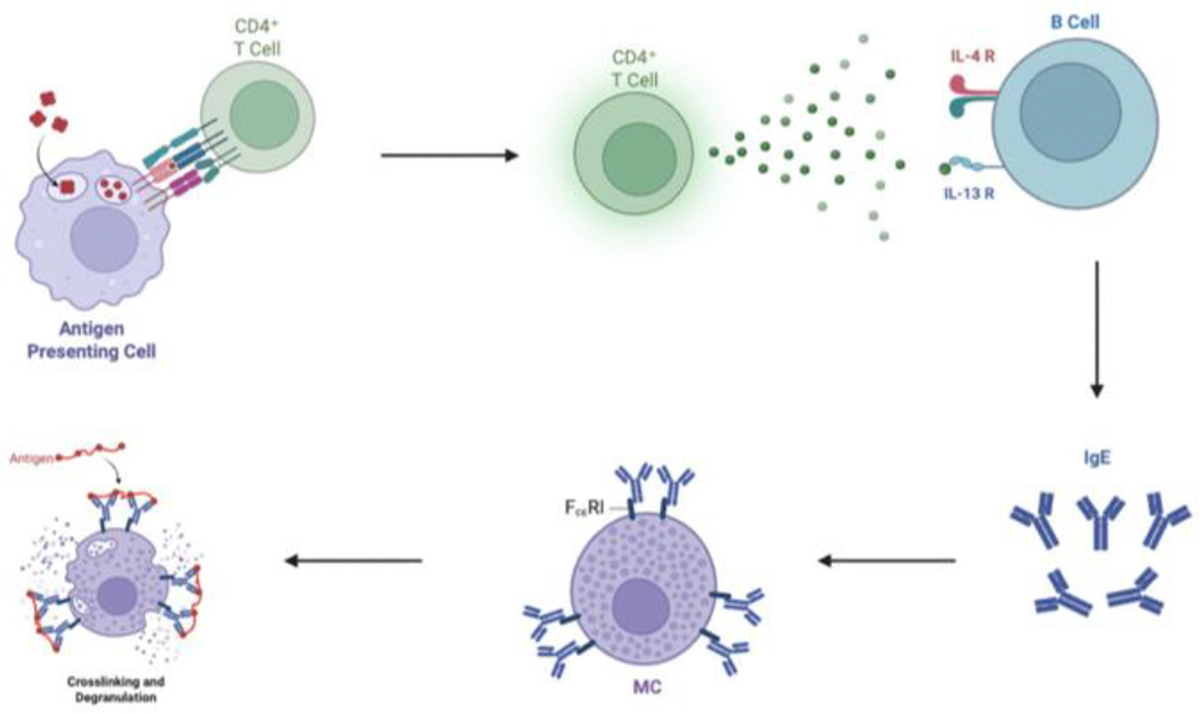
The schematic diagram illustrates the classic Th2-mediated allergic response. First, an antigen-presenting cell uses MHC II to present an antigen to a CD4+ T helper cell, stimulating an IgE-mediated response. The T helper cell then releases cytokines like IL-4 and IL-13, which bind their respective receptors on B cells. This induces isotype switching to produce an abundance of IgE, which then binds to FcεRI on the surface of MCs. Upon future exposure, crosslinking occurs, which elicits a strong response and degranulation of MCs. Created with BioRender.com

IgE-Mediated Activation: IgE-mediated responses are the most-researched aspect of MCs and represent the classical pathway of allergic and antiparasitic immune responses. Since the IgE-mediated response is T cell-dependent, it requires uptake by an antigen-presenting cell, followed by presentation on Major Histocompatibility Complex (MHC) II molecules to T helper (CD4+) cells. This is the priming process responsible for sensitization, during which Th2 cells produce the cytokines IL-4 and IL-13, promoting differentiation and isotype switching to IgE [5,6,7,46]. The resulting IgE antibodies are secreted by B cells into the systemic circulation, where they bind with high affinity to FcεRI on MCs and basophils. FcεRI-anchored IgE binds epitopes of a specific antigen, resulting in a crosslinking process among several IgE molecules on the same MC. The degree of clustering is a determining factor of the strength of intracellular signaling. Experimental studies in mouse models demonstrated that anti-IgE, an experimental crosslinking agent that mimics antigen binding, induced intracellular signaling in MCs [47]. Structurally, FcεRI is a multimeric transmembrane receptor composed of an extracellular α chain that binds IgE, followed by β and γ chains containing immunoreceptor tyrosine-based activation motifs (ITAMs), which initiate downstream signaling. After crosslinking, Lyn kinase phosphorylates the ITAMs on the β and γ chains of FcεRI, thus recruiting Syk for docking. Once activated, Syk kinase stimulates PLCγ to cleave PIP2 into IP3 and DAG, resulting in a significant Ca^2+^ influx [48]. Degranulation occurs when preformed secretory vesicles within MCs are exocytosed due to the increase in intracellular Ca^2+^, resulting in the release of amines, proteases, cytokines, and growth factors into the surrounding interstitial space [49] (Figure 2). The release of these mediators into the surrounding tissue drives inflammatory processes, including vasodilation, increased vascular permeability, nerve stimulation, and smooth muscle contraction, ultimately contributing to the clinical manifestations of allergic and other inflammatory disorders.

## 10. Non-IgE-Mediated Pathways in MCAS

**Figure 3:**
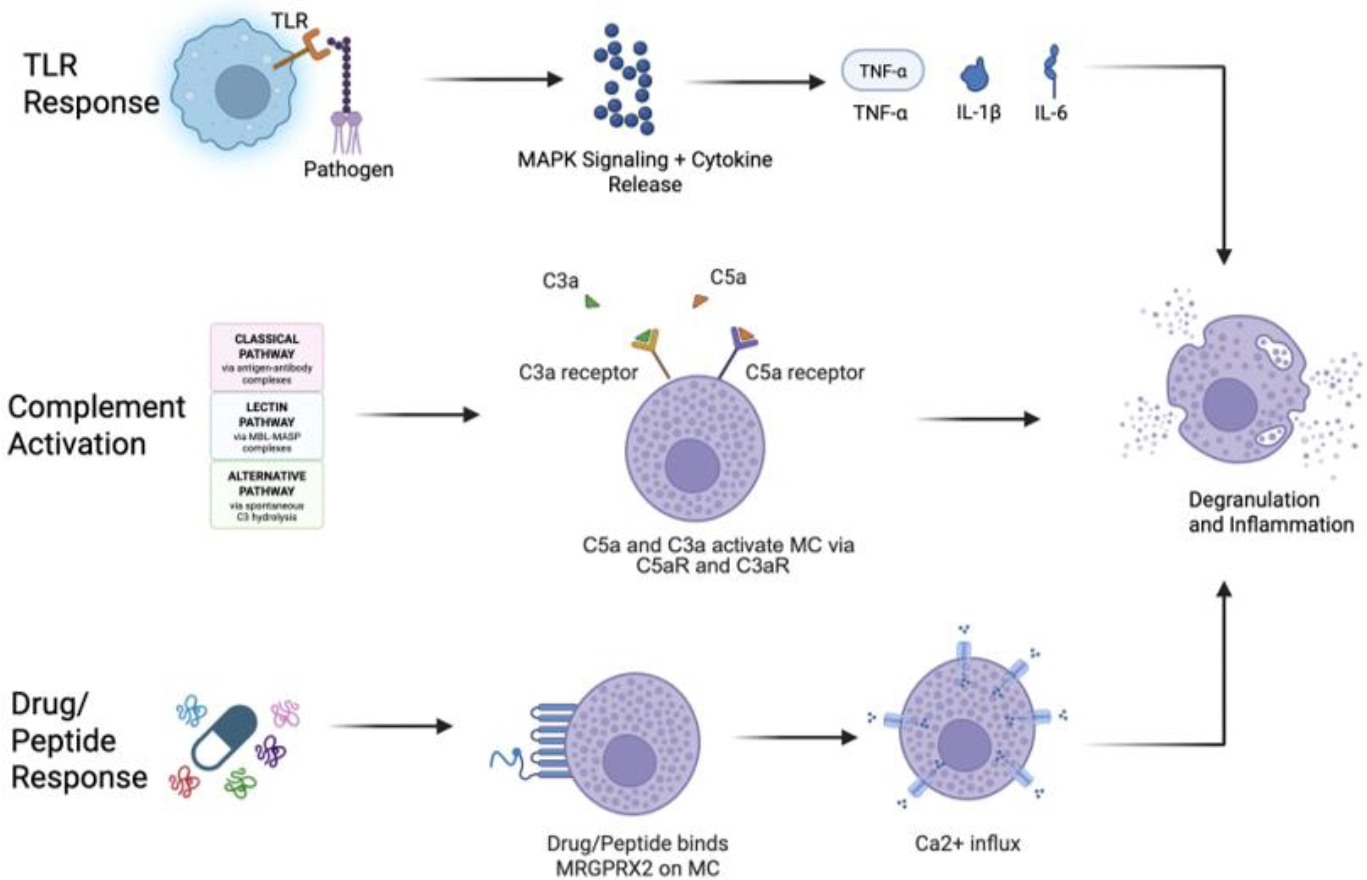
The illustration shows three common pathways of non-IgE-mediated activation of mast cells leading to degranulation and inflammation. The first pathway demonstrated above shows TLR2/TLR4 binding to specific pathogen components, such as bacterial LPS or lipoteichoic acid, thus stimulating MAPK intracellular signaling and the release of cytokines such as TNF-α, IL-6, and IL-1β, resulting in MC degranulation. Next, complement is activated through the classical, lectin, and alternative pathways depending on the initiating stimulus. Following activation, the downstream anaphylatoxins C3a and C5a are generated and bind to MCs, orchestrating degranulation and inflammation. In the final pathway, drugs/endogenous peptides bind to MRGPRX2 on MCs, triggering a massive influx of intracellular Ca^2+^ that results in degranulation and inflammation. Created with BioRender.com

Despite IgE-mediated activation representing the classical and most extensively studied pathway, evidence highlights the importance of IgE-independent mechanisms that may occur concurrently while amplifying MC-mediated pathology (Figure 3). These pathways usually do not require prior sensitization (i.e., an initial priming exposure); instead, they are activated through innate immune signaling via TLRs, complement components such as C3a and C5a, and drug-induced activation through receptors such as Mas-related G protein-coupled receptor X2 (MRGPRX2) [50]. MCs express a variety of TLRs that recognize specific viral and bacterial components; however, TLR4 and TLR2 remain the most well-characterized for their inflammatory effects (Figure 3).

Lipopolysaccharide (LPS), perhaps the best-characterized bacterial endotoxin found on Gram-negative bacteria, is a major activator of TLR4 and stimulates the release of TNF-α, IL-6, and IL-1β, producing a pro-inflammatory response [34,51]. Likewise, TLR2 recognizes Gram-positive bacterial components such as lipoteichoic acid, promoting the release of similar cytokines and further amplifying inflammation.

The complement pathway is a component of the innate immune response consisting of a cascade of plasma proteins that amplify inflammation. There are three main complement pathways based on the initiating stimulus: the classical, lectin, and alternative pathways. Regardless of which pathway is activated, activation of the complement cascade results in the generation of C3a and C5a, which are known as anaphylatoxins. Researchers demonstrated that although IgE-dependent activation is widely recognized as the central cause of reactions, IgE-independent pathways can still elicit a response strong enough to induce anaphylaxis in the absence of IgE [52]. The complement cascade does not just evoke a local response; C3a and C5a are soluble and move throughout the circulation, further activating MCs and contributing to widespread inflammation. Additionally, MCs can also be directly activated through MRGPRX2 by a variety of drugs and endogenous peptides. MRGPRX2 is a GPCR that primarily signals through a Gαq signaling cascade in response to peptidergic drugs, without requiring prior sensitization. MRGPRX2 activation results in pseudoallergic and IgE-independent anaphylactic reactions to pharmacological stimuli while also contributing to MC activation implicated in the pathogenesis of itch and asthma [53]. Experimental studies showed that inhibition of MRGPRX2 significantly reduced the immune response, specifically IgE-independent MC activation [54]. The convergence of multiple activation pathways underscores the ability of MCs to rapidly initiate and amplify inflammatory processes, even in the absence of classical IgE-mediated signaling.

## 11. Genetic and Molecular Factors

Although MCAS is not considered to have a classic Mendelian inheritance pattern, evidence suggests that genetic predispositions contribute to disease development. Researchers have proposed that MCAS and SM may be traced back to genetic alterations involving signaling proteins, intracellular signaling cascades, epigenetic regulation, RNA splicing, and transcription factors. A study of nine families with elevated basal tryptase levels suggested a dominant inheritance pattern within this cohort. Additionally, approximately 75% of patients with SM or MCAS reported having at least one first-degree relative with MCAS [55]. Furthermore, 46% of first-degree relatives were symptomatic, compared with approximately 17% of the general German population [55]. Since the prevalence of MCAS is relatively low and familial data are sparse, this study was particularly noteworthy, illustrating evidence of transgenerational inheritance. Considerable attention has been focused on the multifactorial consequences of somatic mutations in the receptor tyrosine kinase KIT and epigenetic modifications.

KIT is a type III transmembrane receptor tyrosine kinase that is activated by stem cell factors, triggering intracellular signaling pathways that promote MC survival, proliferation, and differentiation. Additionally, KIT plays a critical role in the development and function of hematopoietic progenitor cells, melanocytes, primordial germ cells, and the interstitial cells of Cajal [56]. Cytogenetic analysis identified a specific mutation, KIT(D816V), in exon 17 of the KIT gene, located on chromosome 4q12. This somatic mutation results in ligand-independent activation, consequently leading to the excessive release of MC mediators. Further data indicate that multiple KIT mutations can induce symptoms observed in both MCAS and SM, with KIT(D816V) being the most common. It is important to acknowledge that KIT plays a vital role in the initiation of MC proliferation, but epigenetic mechanisms, including DNA methylation, histone modifications, and microRNAs, also contribute to MC dysregulation and disease susceptibility. Among the various types of non-genetic changes that occur without altering the DNA sequence, histone acetylation and DNA methylation are the most common mechanisms affecting MC reactivity. Histone acetylation involves the addition of an acetyl group to lysine residues on histone proteins, loosening chromatin into a transcriptionally active state known as euchromatin and promoting gene transcription. On the other hand, DNA methylation blocks transcription factor binding sites, thereby inhibiting gene transcription. In cases of hypermethylation by DNA methyltransferases, protective genes, including tumor suppressor genes, may become significantly inactivated [57]. Overall, by turning genes on and off, epigenetic regulation contributes to pathological MC responses and disease susceptibility. The reversible nature of epigenetic modifications presents potential therapeutic targets through the inhibition or activation of methylation and demethylation pathways to help control aberrant MC regulation.

## 12. Intracellular Signaling Cascades

Various ligands including cytokines, allergens, hormones, microbial products, and neurotransmitters activate intracellular signaling cascades which amplify cellular responses and induce transcriptional changes. Major intracellular signaling pathways involved in MC activation are PI3K/Akt/mTOR, RAS/MAPK, and JAK/STAT. The PI3K/Akt/mTOR pathway primarily regulates cell survival, apoptosis, growth, metabolism, and mediator production, making it a key contributor to sustaining MC activation. By prolonging MC activation, this pathway enhances inflammation while exacerbating MCAS dysregulation. The mechanism begins with an extracellular stimulus binding to a cell-surface receptor, initiating PI3K signaling. PI3K then converts PIP2 to PIP3, which recruits and activates Akt. Akt subsequently stimulates mTOR signaling, leading to activation of downstream transcription factors that promote MC survival and growth [58]. An example of this pathway in action is the MC response to the spike protein of severe acute respiratory syndrome coronavirus 2 (SARS-CoV-2), in which researchers observed increased intracellular calcium accumulation and microtubule-dependent granule transport [59]. Following the increase in intracellular Ca^2+^ concentration, degranulation occurs, and results in the release of pro-inflammatory mediators, including histamine, prostaglandins, and cytokines. This study demonstrates that even a relatively subtle stimulus, such as a viral spike protein, can activate MCs. More broadly, these findings highlight the remarkable sensitivity of MCs, helping explain why seemingly minor triggers, including temperature changes, environmental exposures, infections, and other external stimuli, can provoke exaggerated responses in individuals with MCAS. Importantly, MCs integrate signals from a diverse array of triggers simultaneously, and the convergence of these signals on shared intracellular pathways further amplifies immune dysregulation.

Concurrently, the RAS/MAPK pathway functions alongside PI3K/Akt/mTOR signaling to induce MC proliferation, differentiation, and cytokine production. Following receptor activation, intracellular adaptor proteins activate RAS GTPases, initiating a kinase cascade involving RAF (MAPKKK), MEK (MAPKK), and ERK (MAPK), with each kinase phosphorylating the next in line [60]. Next, the activated ERK translocates to the nucleus, where it regulates transcription factors responsible for gene expression. Through this mechanism, the RAS/MAPK pathway promotes MC expansion, maturation, cytokine release, and immune signaling.

Among the highlighted intracellular signaling pathways, JAK/STAT has received considerable attention because of its central role in MC regulation. When cytokines bind to cell-surface receptors, the JAK/STAT pathway is activated, inducing receptor dimerization and subsequent activation of Janus kinases (JAKs). These newly activated JAKs phosphorylate tyrosine residues, creating docking sites that recruit signal transducers and activators of transcription (STATs) to propagate downstream signaling [61]. The STATs dimerize and translocate to the nucleus, where they regulate gene transcription involved in MC survival, proliferation, differentiation, and inflammatory responses. Furthermore, there are several regulatory mechanisms that prevent STAT hyperactivation, including protein tyrosine phosphatases, suppressors of cytokine signaling, and protein inhibitors of activated STATs. These essential negative feedback mechanisms limit excessive JAK/STAT signaling and help protect against the pathological dysregulation associated with MCAS. Among the four members of the JAK family and the seven members of the STAT family, a large retrospective study of patients diagnosed with SM identified elevated levels of phosphorylated JAK2 and STAT5 associated with KIT(D816V) gain-of-function mutations [61]. Elevated phosphorylation of JAK2 and STAT5 in patients with SM underscores the clinical relevance of this pathway. Understanding these key intracellular signaling pathways is essential for the development of targeted therapeutics aimed at mitigating the exaggerated MC responses underlying MCAS.

## 13. Mediator Effects and Tissue Dysfunction

As discussed in the previous sections, many molecular factors and intracellular signaling pathways ultimately result in the release of mediators. These mediators serve as important biomarkers for the diagnosis of MCAS and provide mechanistic insight into why patients experience specific symptoms. MC mediators can be broadly categorized as either preformed or newly synthesized. Preformed mediators are stored within MC granules and released rapidly, typically within seconds to minutes, making them largely responsible for the immediate effects of MC activation. In contrast, newly synthesized mediators are generated following MC activation over minutes to hours through enzymatic pathways, contributing to sustained and chronic inflammatory responses. Understanding the distinct roles of these mediators is essential for explaining how MC activation translates into the widespread tissue dysfunction and systemic manifestations characteristic of MCAS.

## 14. Preformed Mediators

Histamine is an extremely potent preformed mediator responsible for vasodilation, increased vascular permeability, and many of the hallmark symptoms observed in MCAS, including flushing, pruritus, hypotension, and edema. Furthermore, histamine plays a crucial role in the development and maturation of leukocytes and MCs while also promoting their recruitment to sites of allergic reactions and chronic immune responses. Histamine exerts its diverse biological effects through four histamine receptor subtypes (HR1–HR4), each mediating distinct physiological functions. Among the four receptor subtypes, HR1 is a Gαq/11-coupled receptor involved in allergic and inflammatory responses as well as vasodilation through activation of phospholipase C and influx of intracellular Ca^2+^ signaling [62]. HR2 is a Gαs-coupled receptor, which is responsible for gastric acid secretion and relaxation of cardiovascular smooth muscle [62]. HR3 is a Gαi/o-coupled receptor that plays a significant role in the brain and nervous system by regulating wakefulness, cognition, and energy homeostasis [62]. HR4 is a Gαi/o-coupled receptor that promotes immune cell recruitment and inflammatory responses through MAPK activation and increased intracellular Ca^2+^ signaling via autocrine and paracrine mechanisms [62]. As evident through these four main receptor subtypes, histamine plays a key role in the cross-reactivity of multiple physiological systems, helping explain the diverse symptoms experienced by patients with MCAS. Emerging evidence suggests that histamine not only mediates inflammation but also influences MC maturation and phenotype by acting on both the activated MC and neighboring MCs [63]. With the numerous processes in which histamine participates, pharmaceuticals can target these HRs to mitigate symptoms such as pruritus, flushing, and other manifestations of MC dysregulation.

Tryptase, the most abundant granule protein, has a role in the symptoms of MC activation but is more commonly known for its biomarker role in diagnosing immunologic disorders.

Tryptase has a longer half-life (90–120 min) when compared to counterparts such as histamine (1–6 min), making total serum tryptase levels an even more useful diagnostic tool when measured over a period. Mature tryptases cleaved from the inactive precursor protryptase are maintained by heparin proteoglycans, forming tetrameric enzymes that are stored within MC granules alongside other mediators before release [64]. Unlike histamine and its immediate effects, tryptase persists and plays key roles in chronic tissue effects, tissue remodeling, fibrosis, and protease activity [65]. Therefore, although tryptase has a significant role in diagnostics, its involvement in the progression of inflammation illustrates the potential for pharmaceutical interventions that may prove effective.

Heparin, a highly sulfated glycosaminoglycan that is released in granules with histamine, does more than serve as a potent anticoagulant. These proteoglycans play substantial roles in the storage of other granule-stored compounds such as bioactive monoamines (histamine, serotonin, dopamine) and proteases like tryptase [66]. Though not contributing to inflammation and acute effects to the same extent as other mediators, heparin release during degranulation allows organization and longevity among different effectors. Additionally, it could explain the potential for easy bruising or bleeding experienced by some patients. Researchers have reported that plasma heparin levels can also provide a meaningful biomarker for MC activation. Using a large sample of patients diagnosed with MCAS, they concluded that plasma heparin levels have greater sensitivity than other biomarkers, making it particularly useful for screening and detecting exaggerated MC activity [67]. A notable caveat the researchers mentioned was that, although heparin was an extremely valuable biomarker when assessed alone, higher sensitivity and specificity were observed, as anticipated, when it was combined with other biomarkers, illustrating the benefit of evaluating multiple biomarkers during diagnosis [67]. Understanding the mechanisms of these mediators allows various pathways to be targeted through pharmaceuticals, while mediators such as heparin and tryptase also have the potential to provide a more standardized approach to diagnosing conditions such as MCAS.

## 15. Newly Synthesized Mediators

After the release of preformed mediators, leukotrienes and prostaglandins are generated via the metabolism of arachidonic acid, thus contributing to a sustained and amplified inflammatory response. The oxidative metabolism of arachidonic acid is extremely complex, as it can be shunted into either the cyclooxygenase pathway, resulting in two cyclic endoperoxides, PGG2 and PGH2. In patients with mastocytosis, researchers found evidence that these products are converted predominantly to PGD2 by MCs, promoting further recruitment of immune cells [68]. Literature describes PGD2 as an effector molecule involved in the development of chronic pulmonary disease, lung fibrosis, and asthma symptoms. Despite prostaglandins typically being synthesized nonspecifically, PGD2 could potentially be a valuable biomarker, as urinary PGD2 metabolites demonstrate strong correlation with and sensitivity for MC activation [69].

In contrast, arachidonic acid can be converted via 5-lipoxygenase to LTA4, then further to LTB4, LTC4, LTD4, and LTE4 [68]. These pathways do not occur in isolation, nor are they mutually exclusive, as they both promote a pro-inflammatory state and can result in dysregulation, explaining the respiratory symptoms patients experience, such as bronchospasms, as well as dermatologic symptoms resulting from vasodilation. Recognizing its potent contribution to anaphylaxis, researchers have paid particular attention to LTC4. To further highlight the important role of leukotrienes, a sample of mice lacking LTC4 synthase and cysteinyl leukotriene receptors demonstrated a protective effect following MC activation. The mutated mice exhibited reduced plasma leakage, vascular permeability, and fibrosis when compared to the control group [70]. These findings provide compelling evidence that leukotrienes are active contributors to the inflammatory and tissue-remodeling processes characteristic of MCAS.

While many of the previously discussed bioactive mediators directly cause biological effects, cytokines have been briefly alluded to because of their ability to orchestrate downstream inflammatory responses. Cytokines are essential regulators of the immune system, functioning as highly specific signaling molecules that coordinate communication between distinct cell populations. Through receptor-mediated signaling, they influence the location, timing, magnitude, and duration of immune responses. TNF-α (Tumor Necrosis Factor alpha) is known for being the classic MC cytokine, recruiting leukocytes, amplifying inflammation, and increasing endothelial permeability. Furthermore, there is evidence demonstrating that TNF-α may underlie neurological symptoms and headaches through its influence on astrocytes and microglia [71]. In addition to recruiting leukocytes and amplifying inflammation, TNF-α is one of the master cytokines increasing the production of other cytokines, most notably IL-6. IL-6 acts on T cells and B cells while also enhancing mucus production in the airways, hence the respiratory manifestations observed in MCAS [72]. It is especially relevant in MC activation, as it promotes IgE production, leading to chronic inflammatory responses and even the development of systemic inflammation. IL-6 production is influenced by the release of IL-4 but also has similar effects to IL-13 by promoting Th2 and IgE responses associated with allergic reactions and increased MC dysregulation. Although the focus is on cytokines in the context of MCs, they also act on other cells, including macrophages, monocytes, eosinophils, and others. Interestingly, recent literature has suggested that IL-33 is a significant modulator that is not only released by MCs but also acts back on them through a positive regulatory feedback mechanism, creating a vicious self-perpetuating cycle of MC activation. IL-33 has been given the nickname of “alarmin” due to being released during tissue or cellular damage and stress, and in conjunction with other cytokines immunomodulates the cellular response to promote survival and proliferation in many disease conditions [73–77]. IL-33 is unique because it is released early, underscoring its potential as a biomarker while also promoting MC development and maturation through its effects on CD34+ cells. Despite the extraordinary diversity of cytokines, TNF-α, IL-6, IL-4, IL-13, and IL-33 collectively play fundamental roles in MC regulation, driving robust innate and adaptive immune responses.

## 16. Therapeutic Strategies and Pathway Targeting

### First-Line Symptom Control

Recent advances in MCAS treatment have focused on medications that target the mediators responsible for symptom development, with H1 blockers, H2 blockers, and leukotriene receptor antagonists serving as first-line therapies. These therapeutic strategies mitigate symptom burden by targeting many of the signaling pathways and mediators discussed in the previous sections. H1 blockers, including diphenhydramine, cetirizine, fexofenadine, and rupatadine, are particularly effective in alleviating symptoms such as urticaria, flushing, edema, and pruritus. Although there were potential sources of bias, a double-blind, placebo-controlled crossover randomized trial involving 71 patients demonstrated significant improvements in quality of life and pruritus scores. Patients treated with rupatadine had also experienced reductions in symptoms such as itching, flushing, wheeling, flaring, tachycardia, and headaches compared with placebo, despite slight uncertainty in the overall strength of the evidence [16]. Even though there is data supporting the therapeutic utility of H1 blockers, researchers remain reluctant to draw such definitive conclusions, as further research is needed to better establish their efficacy and safety across diverse patient populations. H2 blockers like famotidine and cimetidine have dual roles by alleviating allergic symptoms while improving GI manifestations. The therapeutic application of H2 blockers is like that of H1 blockers, often requiring repeated dosing because their effects are temporary and symptoms reemerge following drug cessation [78]. While the antihistamine therapies often provide meaningful initial symptom improvement, their temporary effects and the recurrence of symptoms highlight the need for additional targeted treatments. Beyond antihistamines, an adjunctive therapy includes montelukast, a leukotriene receptor antagonist that blocks cysteinyl leukotriene receptors and has demonstrated benefit in patients with respiratory symptoms. As articulated, the complex network of mediators involved in MCAS requires distinct therapeutic targets to alleviate the various systemic symptoms caused by the disease, emphasizing that each first-line therapy plays its own unique role in symptom management.

## 17. Mast Cell Stabilizers

As mentioned previously, antihistamines block the effects of histamine by targeting its receptors, whereas MC stabilizers function by increasing the threshold for degranulation to occur, thereby reducing mediator release. Ketotifen is a unique stabilizer because it also possesses antihistamine properties by blocking H1 receptors while primarily functioning to limit MC degranulation. Although the precise mechanism of action of ketotifen remains incompletely understood, current data suggest it is effective in patients with MC-mediated diseases. Ketotifen therapy was associated with a reduction of circulating plasma histamine levels and inhibition of cutaneous MC degranulation in individuals with cold-induced urticaria [79]. To further emphasize the effectiveness of ketotifen in diminishing the inflammatory response, a rat model of gout characterized by recurrent inflammatory attacks demonstrated meaningful reductions in inflammation. Specifically, significantly lower levels of pro-inflammatory mediators, including nitric oxide, IL-1β, and IL-6, were observed in the ketotifen-treated group compared with controls [80]. Collectively, these findings indicate that ketotifen not only improves histamine-mediated symptoms such as urticaria but also exerts broader anti-inflammatory effects by reducing pro-inflammatory cytokines and nitric oxide. By targeting both histamine signaling and MC degranulation, ketotifen bridges traditional antihistamine therapy with MC stabilization through its dual mechanisms of action.

Cromolyn sodium is one of the most widely utilized MC stabilizers and provides significant symptom control for patients with MCAS. Cromolyn has been used for decades, is generally well tolerated, and has relatively mild adverse effects. Originally extracted from the eastern Mediterranean herb Ammi visnaga, cromolyn was initially used to treat chronic asthma because of its ability to reduce bronchial hyperreactivity. Over the years, it has demonstrated significant efficacy in inhibiting MC mediator release [81]. Like ketotifen, the precise mechanism of action of cromolyn has not been fully elucidated, but substantial evidence supports its ability to inhibit downstream signaling pathways leading to the release of histamine, leukotrienes, and other potent MC mediators [82]. Over the years, cromolyn's clinical indications have expanded to include allergic rhinitis, MCAS, mastocytosis, asthma, allergic eye conditions, and nasal allergies. It also improves symptoms such as flushing, headache, abdominal pain, nausea, diarrhea, and vomiting in patients with MCAS [83]. Cromolyn sodium has been a crucial addition to the pharmaceutical repertoire by targeting mechanisms involved in MC activation while also mitigating symptoms associated with MCAS and other immunologic disorders. Although further research is needed to better understand its mechanism of action, current evidence remains encouraging. When combined with other first-line therapies, MC stabilizers may provide enhanced symptom control by targeting both mediator release and mediator activity.

## 18. Targeting Signaling Pathways

Although omalizumab is not classified as a traditional MC stabilizer, it similarly reduces MC activation and subsequent degranulation through a distinct mechanism. Omalizumab is a monoclonal anti-IgE antibody that binds free IgE, thus decreasing its availability to bind FcεRI on MCs and basophils, resulting in reduced activation of IgE-dependent pathways. Omalizumab is recommended for patients 12 years of age or older with chronic spontaneous urticaria whose symptoms persist even when treated with high-dose antihistamines [84]. With the success observed in patients with urticaria and asthma, omalizumab has also been used off label for symptom control in MCAS. Akin summarized a 2020 systematic review published in the Journal of Allergy and Clinical Immunology, which included two retrospective cohort studies and several case series comprising a total sample of 69 patients. The review reported an 84% rate of complete resolution of anaphylaxis, along with improvements in palpitations, GI symptoms, and cutaneous manifestations [85]. Although these results are promising, the review emphasized that evidence across studies remains inconsistent, with several reports showing less pronounced symptom improvement, suggesting the possibility that these findings may represent an outlier within the current literature. Clinical guidelines recommend omalizumab as a second-line therapy after antihistamines, with cyclosporine reserved as a third-line treatment for refractory cases.

KIT has emerged as a promising therapeutic target for MCAS and related MC disorders because of its central role in MC survival, activation, and proliferation. Specifically, the KIT(D816V) mutation promotes constitutive KIT signaling, resulting in MC hyperproliferation and enhanced survival. Therefore, targeted pharmaceuticals such as tyrosine kinase inhibitors (KIT inhibitors) have been developed to limit disease progression. Imatinib was the first KIT inhibitor developed and demonstrated efficacy against most KIT mutations, although it lacks activity against KIT(D816V) [86]. Subsequently, dasatinib was developed and, despite its relatively short half-life, retained activity against both the KIT(D816V) mutation and wild-type KIT [86]. Lastly, midostaurin was originally developed as a protein kinase C inhibitor and later found to possess multikinase activity, including inhibition of KIT(D816V). This broad spectrum of kinase inhibition affects pathways involved in the cell cycle, oncogenic signaling, and additional intracellular signaling cascades [86]. As articulated, there are well-established KIT inhibitors that can be beneficial for patients with disorders due to MC dysregulation; however, mutations such as KIT(D816V) continue to warrant further development and research. Among emerging KIT inhibitors is the thiazole amine compound designated "126332." This compound has shown encouraging results by inhibiting KIT phosphorylation, which in turn suppresses downstream STAT5 signaling [87]. Furthermore, this compound downregulated the anti-apoptotic proteins survivin and Mcl-1, thereby inducing MC apoptosis.

Given the pivotal role of JAK/STAT signaling in MC activation, this pathway has become an attractive target for therapeutic intervention. The specific involvement of JAK2-STAT5 in MC survival and proliferation has piqued interest in JAK1/JAK2 inhibitors such as ruxolitinib. Ruxolitinib has been observed to effectively decrease MC degranulation and the production of pro-inflammatory cytokines, including IL-6, TNF-α, and CCL2. Furthermore, ruxolitinib has shown efficacy in alleviating symptoms such as pruritus and fatigue in patients with JAK2-driven myeloproliferative disorders [88]. Additional research on ruxolitinib revealed that it also inhibits the activation of both MCs and basophils. In a study using human basophils and skin MCs, ruxolitinib exhibited concentration-dependent inhibition of the IgE-mediated release of the preformed mediators, histamine, tryptase, and chymase, as well as leukotriene C4 [89]. Interestingly, the authors also suggested that ruxolitinib inhibits IL-3-mediated release of the cytokines IL-4 and IL-13 from basophils. Overall, the multitude of downstream targets disrupted by ruxolitinib underscores its therapeutic potential for patients with mastocytosis and MCAS while also opening new avenues for development of additional therapies targeting other dysregulated signaling pathways.

Bruton's tyrosine kinase (BTK) is a crucial signaling molecule in FcεRI-mediated MC activation that also mediates signaling through B-cell receptors, TLRs, and chemokine receptors. Studies suggest that BTK inhibitors represent a potential strategy to attenuate MC activation, histamine release, cytokine production, and downstream immune responses. BTK inhibitors such as brutinib, acalabrutinib, and zanubrutinib are known to bind covalently and irreversibly to Cys481, thereby inhibiting BTK phosphorylation and downstream signaling while exhibiting similar pharmacokinetic and pharmacodynamic properties [90]. Ibrutinib was the first BTK developed in 2013 and has demonstrated a potent therapeutic effect in IgE responses. More recently, acalabrutinib and zanubrutinib have been developed for a more selective and safer chemical, with fewer off-target effects [59]. Collectively, these pharmaceutical compounds show potential in diminishing MC degranulation and modulating numerous dysregulated signaling pathways that influence MC function. Furthermore, additional BTK inhibitors, including remibrutinib and fenebrutinib, have shown encouraging results and are currently undergoing further clinical evaluation.

## 19. Anti-inflammatory Strategies

Corticosteroids like prednisone and methylprednisolone remain effective for anti-inflammatory therapy in patients experiencing severe MCAS symptoms and acute disease exacerbations. Unlike antihistamines and MC stabilizers, corticosteroids function as non-specific immunosuppressive agents by suppressing multiple pro-inflammatory pathways and mediators. Corticosteroids’ mechanism of action is to suppress the production and activity of pro-inflammatory cytokines, including TNF-α, IL-1β, and IL-6, to reduce MC activation and improve the multisystem symptoms. In a study with 12 randomized trials that enrolled 944 patients, corticosteroids improved urticaria activity and pruritus but were associated with a 15% increase in adverse effects [91]. While corticosteroids provide effective short-term relief, their adverse effects pose a risk for long-term treatment. Corticosteroids remain a valuable therapeutic option despite adverse effects such as GI irritability, headaches, and anxiety. These risks attest to the need for more specifically targeted therapeutic approaches to minimize systemic toxicity.

Since vitamin D is an immunomodulator and its deficiency could result in the pathogenesis of several inflammatory conditions, including allergic disease and asthma, vitamin D supplementation has been found to be effective in controlling the clinical symptoms due to mast cell activation and other immunological responses in allergies, asthma, atopic dermatitis, and other inflammatory diseases [92–99].

In populations without access to omalizumab, cyclosporine serves as an alternative therapeutic agent that acts as a T-cell immunosuppressant by inhibiting cytokine release from MCs and basophils. Despite demonstrating a 73% response rate in patients with chronic urticaria in both a meta-analysis and a placebo-controlled study, cyclosporine was associated with adverse effects in approximately 50% of patients, particularly renal complications [100]. Although cyclosporine has demonstrated clinical benefit, its substantial risk of adverse effects limits its use, explaining why it is reserved as a third-line therapy after antihistamines and omalizumab.

## 20. Lifestyle and Trigger Avoidance

Management of affected individuals should follow a personalized approach, beginning with less invasive interventions such as lifestyle modifications and trigger avoidance before utilizing antihistamines and eventually other medications. The patient-centered philosophy recognizes that no two patients with MCAS present identically, reflecting the variability in clinical manifestations associated with the disease. Initially, one of the most frustrating aspects of MCAS is identifying the trigger, which often requires trial and error to determine whether symptoms are related to an individual's diet, environmental factors, or psychological stress. Commonly reported triggers include specific foods, medications, environmental exposures, temperature changes, infections, exercise, and psychological stress. This underscores the value of both patient self-awareness and the necessity for physicians to holistically evaluate all aspects of a patient's life to determine the cause of their symptoms. Although solely avoiding precipitating factors may not eliminate symptoms, identifying and minimizing known triggers can significantly reduce the chance of recurrent disease. In combination with the pharmacologic therapies, this approach can further improve quality of life.

## Conclusion

MCAS imposes a substantial burden on affected individuals and commonly disrupts multiple organ systems. This often means that MCAS does not present the same way in all patients, thus making it very difficult to diagnose and treat. This review provided insight into the frequently reported symptoms experienced by patients, including dermatologic, GI, respiratory, neurological, and cardiovascular complaints. The physiology of MC activation is complex, and the dysregulation of other immune cells makes the process far more challenging to understand. Although MCAS remains a complex disease to diagnose and understand, research has identified specific intracellular pathways, molecular factors, and mediators that contribute to disease pathogenesis and may serve as potential therapeutic targets. Before initiating pharmacologic therapies, the first step in managing patients with MCAS should be to identify and eliminate the trigger, whether it is food, environmental factors, or psychological stress. The first pharmaceutical therapy discussed was antihistamines, which block the receptors of the potent mediator histamine. The next line of therapy includes MC stabilizers, intracellular inhibitors, and corticosteroids, which can be used in addition to or as replacements for previous treatments. Unfortunately, these therapies are not without consequences, as many are associated with adverse effects that must be carefully weighed against their potential benefits.

This review highlights numerous promising treatment strategies, but significant opportunities for future research remain. A major limitation of the treatments discussed is that they primarily target symptom management and MC control rather than the root cause of the disease. As advancements in immunology and allergy research continue, there is hope that identifying additional molecular and genetic factors of MCs will lead to more precise disease-modifying therapies. Alongside the challenges of treatment, the diagnosis of MCAS also remains elusive because there is no singular biomarker that can definitively diagnose the disease. Some biomarkers with supporting evidence include serum tryptase, histamine, prostaglandin D_2_, leukotriene E_4_, and heparin, but these are often time-sensitive and lack sufficient sensitivity and specificity. Another challenge is the lack of standardized diagnostic criteria has resulted in inconsistent diagnostic practices, where a patient may receive an MCAS diagnosis at one clinic but not another. Overall, this review has presented a comprehensive overview of MCAS, and there remains optimism for future discoveries. Continued development of cutting-edge technologies and high-quality clinical studies will be essential for advancing effective disease-modifying therapies that can be personalized for patients.

## Key Points

- MCAS is a complex, underrecognized, heterogeneous multisystem disorder characterized by dysregulated MC activation, resulting in diverse dermatologic, GI, respiratory, neurological, and cardiovascular manifestations.
- Activation of MCs can occur through both classical IgE/FcεRI-mediated pathways involving adaptive immunity and non-IgE pathways mediated by TLRs, complement proteins, and MRGPRX2 signaling.
- Abnormal MC activation is driven by a combination of genetic and molecular factors, including types of KIT mutations, epigenetic modifications, and dysregulation of several intracellular signaling pathways.
- PI3K/Akt/mTOR, RAS/MAPK, and JAK/STAT are essential intracellular signaling pathways that regulate MC survival, proliferation, and inflammatory responses which make them attractive targets for pharmacologic interventions.
- Many mediators released by MCs play crucial roles in inflammation such as histamine, tryptase, heparin, prostaglandins, leukotrienes, and cytokines. They are responsible for contributing to the systemic clinical manifestations of MCAS along with serving as clinical diagnostic biomarkers.
- First-line treatment strategies block the receptors of mediators like H1/H2 antihistamines and leukotriene receptor antagonists. While MC stabilizers provide symptom relief by limiting mediator release.
- Targeted therapies such as omalizumab, KIT inhibitors, JAK inhibitors, and BTK inhibitors modulate significant intracellular cascades that are involved in MC activation.
- Corticosteroids and cyclosporine may be beneficial for severe or refractory disease, but their long-term use is often limited by risk of adverse effects.
- Individualized lifestyle modifications and trigger avoidance remain the cornerstone of MCAS management and pharmacologic therapies can be implemented alongside these initial strategies.
- Future research should focus on identifying more reliable diagnostic biomarkers, establishing standardized diagnostic criteria, and developing individualized disease-modifying therapies that improve long term quality of life for individuals suffering from MCAS.
